# Roles of Intramolecular and Intermolecular Hydrogen Bonding in a Three-Water-Assisted Mechanism of Succinimide Formation from Aspartic Acid Residues

**DOI:** 10.3390/molecules190811440

**Published:** 2014-08-04

**Authors:** Ohgi Takahashi, Ryota Kirikoshi, Noriyoshi Manabe

**Affiliations:** Tohoku Pharmaceutical University, 4-4-1 Komatsushima, Aoba-ku, Sendai 981-8558, Japan; E-Mails: kirikoshi@tohoku-pharm.ac.jp (R.K.); manabe@tohoku-pharm.ac.jp (N.M.)

**Keywords:** aspartic acid residue, nonenzymatic reaction, succinimide intermediate, density functional theory, water catalysis, multiple proton transfer, amide-iminol tautomerism

## Abstract

Aspartic acid (Asp) residues in peptides and proteins are prone to isomerization to the β-form and racemization via a five-membered succinimide intermediate. These nonenzymatic reactions have relevance to aging and age-related diseases. In this paper, we report a three water molecule-assisted, six-step mechanism for the formation of succinimide from Asp residues found by density functional theory calculations. The first two steps constitute a stepwise iminolization of the C-terminal amide group. This iminolization involves a quintuple proton transfer along intramolecular and intermolecular hydrogen bonds formed by the C-terminal amide group, the side-chain carboxyl group, and the three water molecules. After a conformational change (which breaks the intramolecular hydrogen bond involving the iminol nitrogen) and a reorganization of water molecules, the iminol nitrogen nucleophilically attacks the carboxyl carbon of the Asp side chain to form a five-membered ring. This cyclization is accompanied by a triple proton transfer involving two water molecules, so that a *gem*-diol tetrahedral intermediate is formed. The last step is dehydration of the *gem*-diol group catalyzed by one water molecule, and this is the rate-determining step. The calculated overall activation barrier (26.7 kcal mol^−1^) agrees well with an experimental activation energy.

## 1. Introduction

**Scheme 1:**
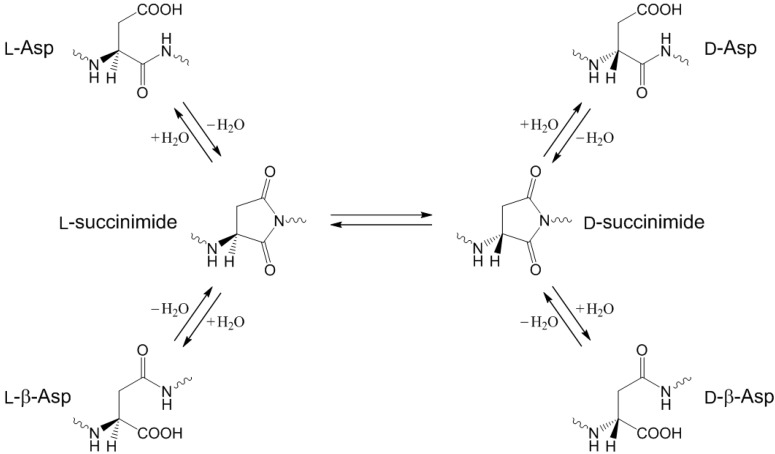
Interdependent isomerization and racemization reactions of l-Asp residues in peptides and proteins via the succinimide intermediate.

Aspartic acid (Asp or D) residues in peptides and proteins (l-Asp) are prone to isomerization to the β-form and racemization under physiological conditions [1,2,3]. These reactions occur spontaneously (*i.e.*, nonenzymatically) and interdependently via a cyclic intermediate (succinimide) as shown in Scheme 1, giving rise to the biologically uncommon l-β-Asp, d-Asp, and d-β-Asp residues. The formation of these altered Asp residues in proteins, which can affect their three-dimensional structures and hence their properties and functions, has indeed been shown to have close relevance to aging and pathologies (especially those of age-related diseases such as cataracts and Alzheimer’s disease) [3,4,5,6,7,8,9,10]. Although the succinimide-mediated reactions of the Asp residues have been studied extensively since the pioneering work by Geiger and Clarke [1], many mechanistic details remain to be clarified.

**Scheme 2:**
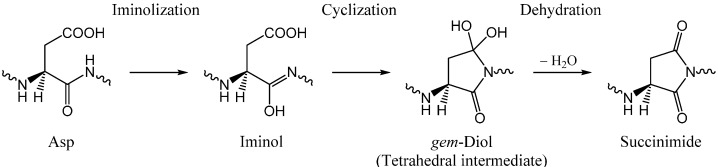
The iminolization−cyclization−dehydration mechanism of the succinimide formation from an Asp residue.

Our recent computational studies [11,12] have shown that, under physiological conditions, it is quite likely that the succinimide formation reactions from Asp residues are catalyzed by two or three water molecules. Moreover, we have shown that the reactions are likely to be initiated by iminolization (*i.e.*, isomerization to the iminol tautomer) of the amide group on the C-terminal side of the Asp residue. The succinimide intermediates are usually thought to result from nucleophilic attack by the C-terminal amide nitrogen on the side-chain carboxyl carbon. However, amide nitrogens are generally poor nucleophiles. On the other hand, iminol nitrogens are expected to be much better nucleophiles [11,12]. We have thus proposed the novel iminolization−cyclization−dehydration mechanism as shown in Scheme 2.

However, our previous computational studies have dealt with the reaction pathway up to the *gem*-diol tetrahedral precursor of the succinimide product and not with the last dehydration step. Catak *et al.* [13] have computationally investigated a one-water-assisted mechanism where direct cyclization from the amide form to give the *gem*-diol intermediate is followed by dehydration. However, the calculated activation barrier of 46.1 kcal mol^−1^ is too high for a biological reaction.

In the present paper, we report new computational results which provide, for the first time, a qualitatively satisfactory reaction model of the *entire* reaction from Asp to succinimide. The reaction is assisted by three water molecules, but in a different manner from the previously proposed three water molecule-assisted mechanism [12]. In particular, the first two steps, which constitute a stepwise iminolization process, involve a quintuple proton transfer along intramolecular and intermolecular hydrogen bonds. This iminolization process is similar, except for the stepwise nature, to the previously reported two water molecule-catalyzed concerted mechanism [11].

## 2. Results and Discussion

**Figure 1:**
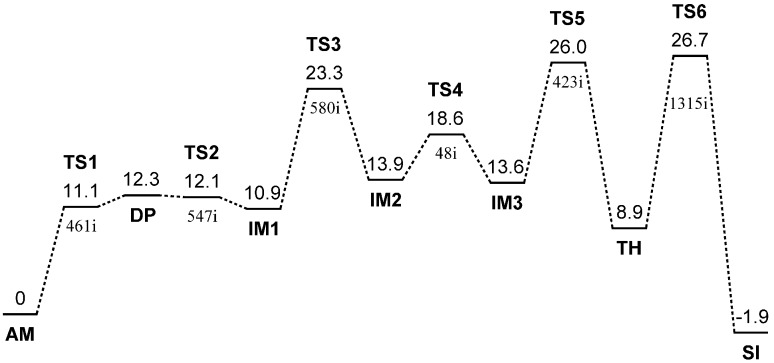
Energy diagram where the ZPE-corrected relative energies (kcal mol^−1^) with respected to **AM** are shown for all the optimized geometries (the total energy of **AM** is −913.830022 *E*_h_). For abbreviations of the optimized geometries, see text. The single imaginary frequency (cm^−1^) is also shown for each TS geometry.

A six-step reaction pathway was found starting from the reactant complex **AM** (amide form). Figure 1 shows the energy diagram for the entire reaction from **AM** to the succinimide product **SI**.

**Figure 2:**
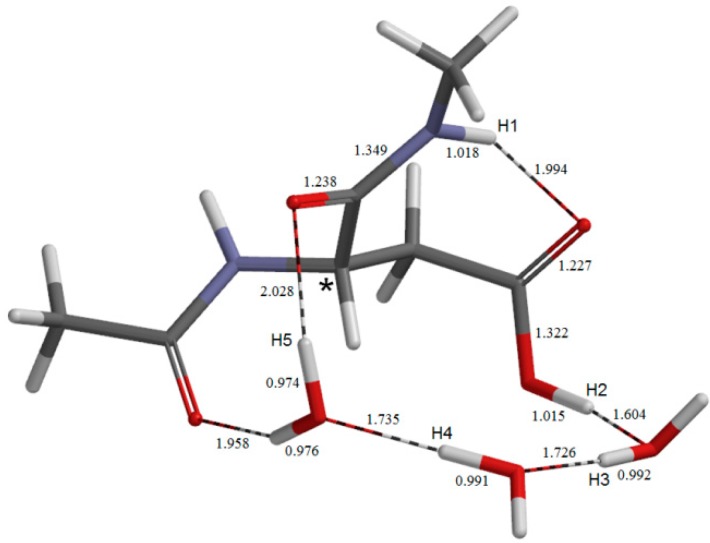
The geometry of the reactant complex **AM**. The α carbon atom is indicated by an asterisk. Selected bond distances are shown in Å.

**Figure 3:**
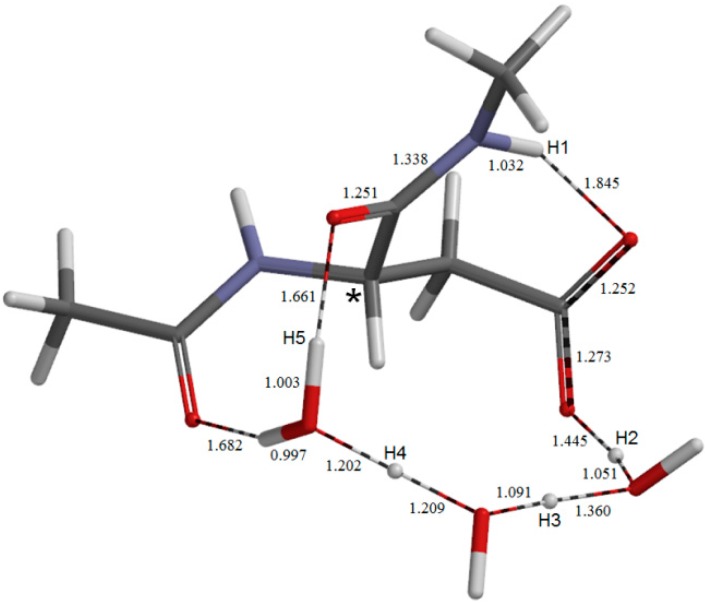
The geometry of the first-step transition state **TS1**. Selected interatomic distances are shown in Å.

**Figure 4:**
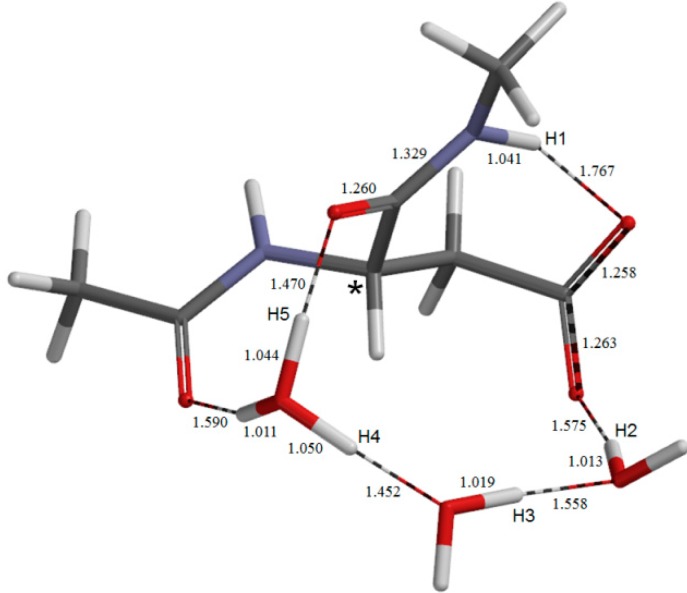
The geometry of the first intermediate **DP**. Selected bond distances are shown in Å.

**Figure 5:**
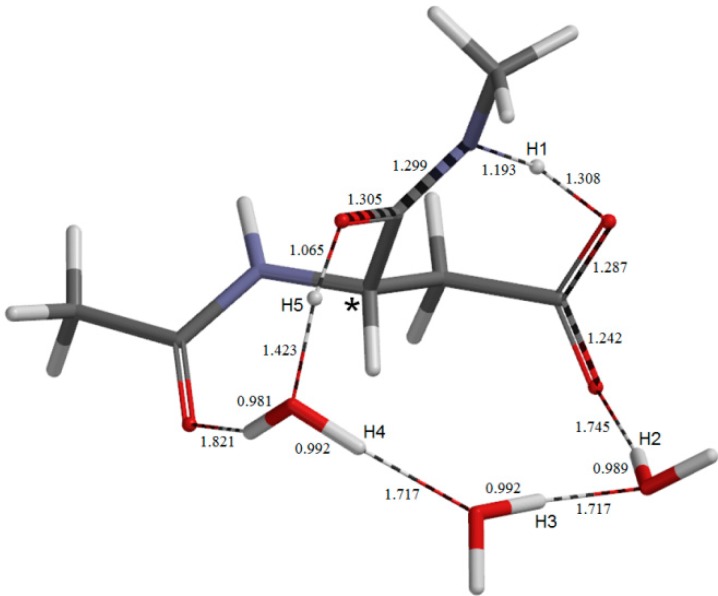
The geometry of the second-step transition state **TS2**. Selected interatomic distances are shown in Å.

**Figure 6:**
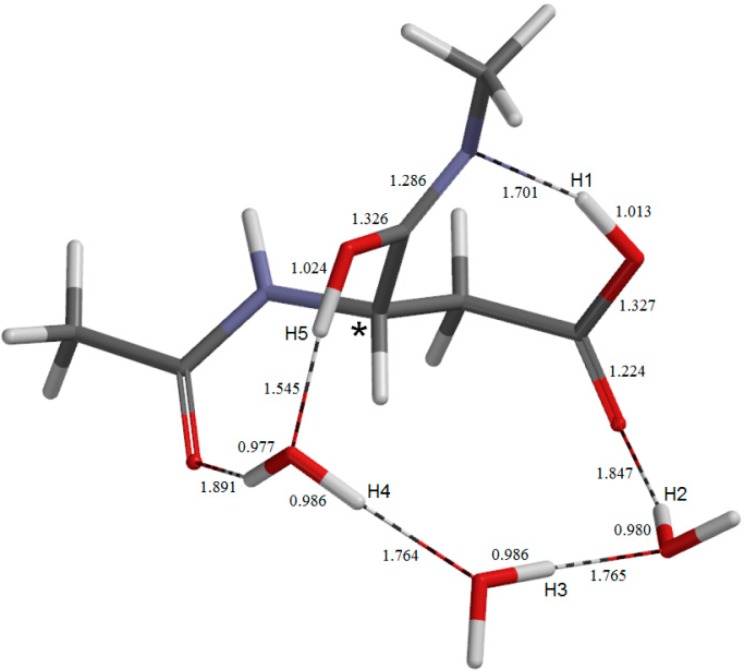
The geometry of the second intermediate **IM1**. Selected bond distances are shown in Å.

**Figure 7:**
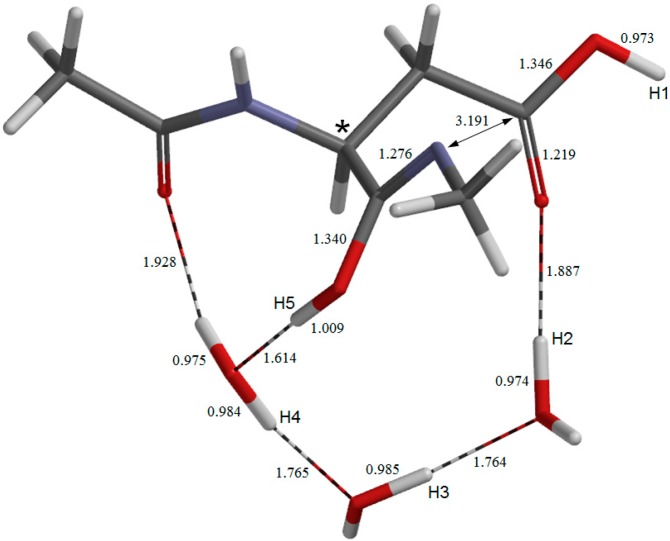
The geometry of the third intermediate **IM2**. Selected bond distances are shown in Å.

**Figure 8:**
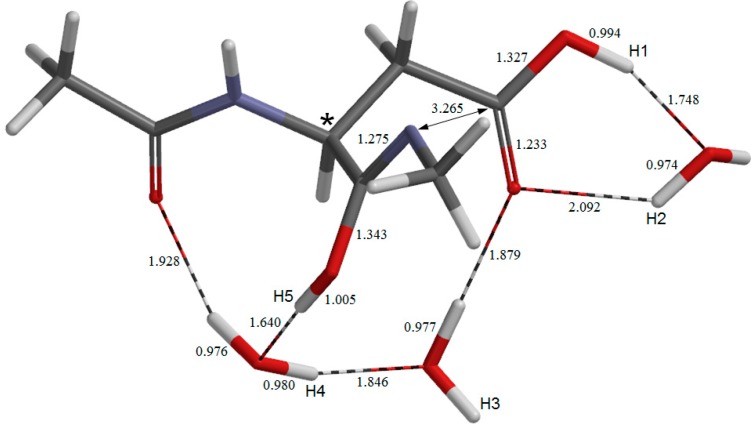
The geometry of the fourth intermediate **IM3**. Selected bond distances are shown in Å.

**Figure 9:**
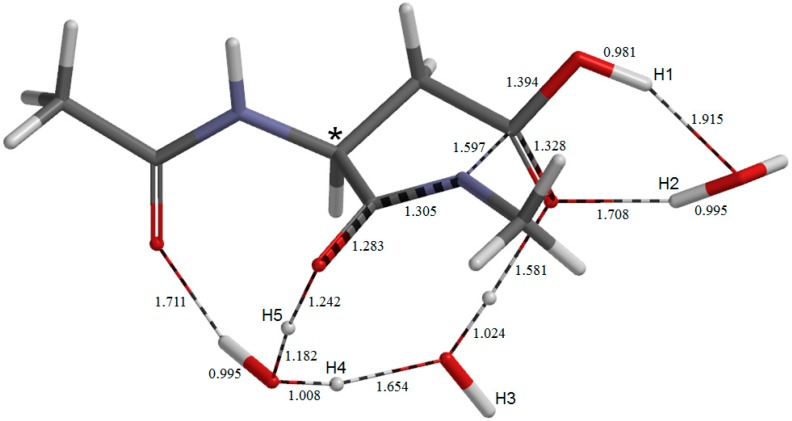
The geometry of the fifth-step transition state **TS5**. Selected interatomic distances are shown in Å.

**Figure 10:**
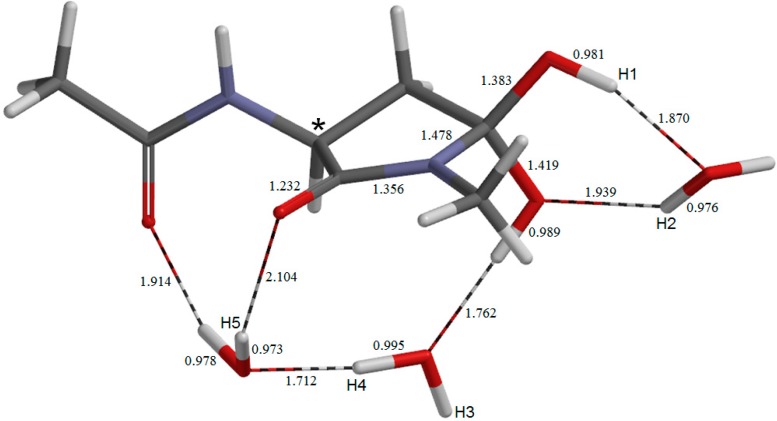
The geometry of the fifth intermediate **TH**. Selected bond distances are shown in Å.

**Figure 11:**
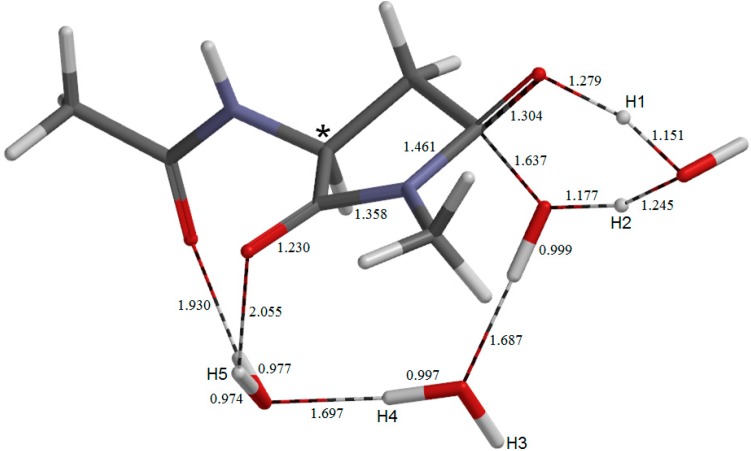
The geometry of the sixth-step transition state **TS6**. Selected interatomic distances are shown in Å.

**Figure 12:**
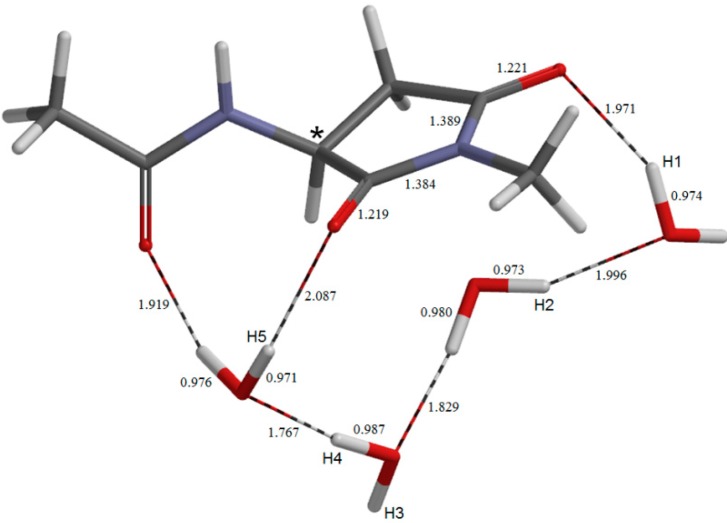
The geometry of the succinimide product **SI**. Selected bond distances are shown in Å.

**Figure 13:**
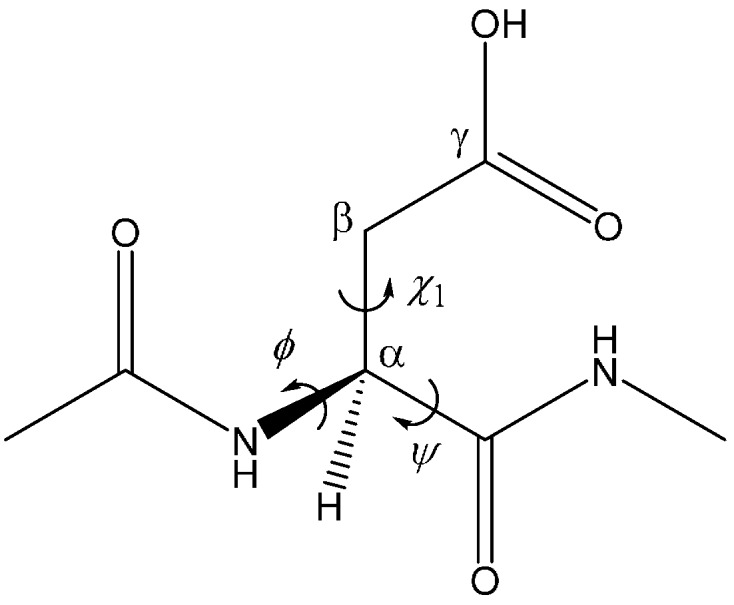
The model compound used in the present study (Ace−Asp−Nme). The *φ* (C−N−C_α_−C) and *ψ* (N−C_α_−C−N) dihedral angles which characterize the backbone conformation and the *χ*_1_ dihedral angle (N−C_α_−C_β_−C_γ_) which characterizes the side-chain conformation are indicated.

Note that **SI** is a complex including four water molecules because a water molecule is released in the last dehydration step. The six TSs are numbered consecutively and abbreviated as **TS1**, **TS2**, **TS3**, **TS4**, **TS5**, and **TS6**. **DP** stands for the deprotonated form in which the side-chain carboxyl group is in the dissociated form (-COO^−^). **IM1**, **IM2**, and **IM3** are complexes between the iminol form and the three water molecules, which differ in conformation and complexation mode. **TH** stands for the *gem*-diol tetrahedral intermediate (complexed with the three water molecules). The optimized geometries (except for **TS3** and **TS4**) are shown in Figure 2, Figure 3, Figure 4, Figure 5, Figure 6, Figure 7, Figure 8, Figure 9, Figure 10, Figure 11 and Figure 12, respectively. Table 1 lists the *φ*, *ψ*, and *χ*_1_ dihedral angles (Figure 13) of all the optimized geometries.

**Table 1:** The *φ*, *ψ*, and *χ*_1_ dihedral angles (°) (Figure 13) of the optimized geometries.

Geometry	*φ*	*ψ*	*χ*_1_
**AM**	−101	−133	−162
**TS1**	−102	−126	−166
**DP**	−103	−121	−171
**TS2**	−112	−118	−179
**IM1**	−116	−109	−173
**TS3**	−121	−110	173
**IM2**	−122	−98	−173
**TS4**	−115	−98	−174
**IM3**	−114	−93	−173
**TS5**	−97	−136	155
**TH**	−97	−142	151
**TS6**	−96	−146	153
**SI**	−100	−134	136

### 2.1. Iminolization

The geometry of the reactant complex **AM** is shown in Figure 2, where five hydrogen atoms relevant to the following discussion are labeled as H1, H2, H3, H4 and H5. The *φ*, *ψ*, and *χ*_1_ dihedral angles of **AM** are −101°, −133°, and −162°, respectively (Table 1). In **AM**, a wire of the three water molecules forms a hydrogen-bond bridge between the backbone carbonyl oxygen and the side-chain OH hydrogen (H2) of the Asp residue. There is also an intramolecular hydrogen bond (1.994 Å) between the NH hydrogen (H1) of the C-terminal amide group and the side-chain C=O oxygen. Thus, there exists a large cyclic structure formed by the C-terminal amide group, the side-chain carboxyl group, and the three water molecules. Moreover, one water molecule (the left one in Figure 2) also forms an additional hydrogen bond (1.958 Å) to the oxygen of the acetyl group (which mimics the neighboring residue on the N-terminal side).

The first two steps in Figure 1 constitute a stepwise iminolization from **AM** to give **IM1** (Figure 6). **TS1** (Figure 3) and **TS2** (Figure 5) are the TSs of the first and second steps, respectively, and **DP** (Figure 4) is the intermediate lying between **TS1** and **TS2**. In the first step, a triple proton transfer involving H2, H3, and H4 occurs to give **DP**. The side-chain carboxyl group (-COOH) becomes deprotonated (-COO^−^) by this step. Concomitantly, the left water molecule in Figure 2 is protonated leading to the formation of an oxonium ion H_3_O^+^. As may be seen from Figure 3, this triple proton transfer is asynchronous (though concerted); more specifically, transfers of H2 and H3 are followed by that of H4. In the second step, a concerted double proton transfer to give **IM1** occurs. More specifically, H1 is transferred to the COO^−^ group along the intramolecular hydrogen bond, and H5 in the H_3_O^+^ ion is transferred to the oxygen of the C-terminal amide group. This double proton transfer is also asynchronous; transfer of H5 precedes that of H1, and so **TS2** possesses an ‘O-protonated amide group’. No other pathways connecting **AM** and **IM1** were found.

Energetically, the intermediate **DP** corresponds to a very shallow minimum on the potential energy surface. Indeed, after the ZPE correction, its energy becomes even slightly higher than those of **TS1** and **TS2** as shown in Figure 1. Before the ZPE correction, the energies of **TS1** and **TS2** are higher than **DP** by 0.97 and 2.05 kcal mol^−1^, respectively. It may be said that **TS1**, **DP**, and **TS2** constitute a very broad, plateau-like transition state ‘region’ [14]. The ZPE-corrected relative energies of **DP** and **IM1** with respected to **AM** are 12.3 and 10.9 kcal mol^−1^, respectively. Although the backward reaction from **IM1** to **AM** is almost barrierless, it is rather surprising that the iminol tautomer can be said to be energetically accessible under the physiological temperature of 37 °C. The hydrogen bond involving the acetyl oxygen becomes very short (1.590 Å) in **DP** (Figure 4), indicating that it plays an important role in stabilizing the H_3_O^+^ ion and hence **DP**. It is also interesting to note that the hydrogen bond involving the iminol hydrogen (H5) is considerably short (1.545 Å, Figure 6). Short hydrogen bonds involving iminol hydrogens were also noted in previous computational studies [11,12]. Moreover, the changes in the *φ*, *ψ*, and *χ*_1_ dihedral angles on going from **AM** to **IM1** are modest (Table 1) (the largest change by 24° is observed in *ψ*). This may suggest that the iminolization by the three water molecule-catalyzed mechanism as shown here can occur even in a structurally-constrained region in a peptide chain.

### 2.2. Change in Conformation and Complexation Mode in the Iminol Form

It seems that a direct cyclization from **IM1** is inhibited by the intramolecular hydrogen bond between the iminol nitrogen and the side-chain carboxyl hydrogen (H1) both structurally and electronically [11]. This hydrogen bond is broken by the *trans*-to-*cis* conformational change in the carboxyl group (O=C−O−H), *i.e.*, rotation about the C−O single bond. This occurs via **TS3** (geometry not shown) with a somewhat high local activation barrier (12.4 kcal mol^−1^). The resulting **IM2** (Figure 7) is higher in energy than **IM1** by 3 kcal mol^−1^. The O=C−O−H dihedral angles in **IM1**, **TS3**, and **IM2** are −166°, −83°, and 0°, respectively. Also notable is that rotation about the C_β_−C_γ_ bond strongly couples with the OH rotation; the C_α_−C_β_−C_γ_=O dihedral angles in **IM1**, **TS3**, and **IM2** are 102°, 51°, and 18°, respectively.

**IM2** is converted to the third iminol minimum **IM3** (Figure 8) via **TS4** (geometry not shown) with a local activation barrier of 4.7 kcal mol^−1^. In this conversion, one water molecule (the right one in Figure 7) moves to a new position where it forms two hydrogen bonds with the carboxyl group (see Figure 8). This water molecule acts later as a catalyst in the dehydration of the *gem*-diol species. **IM3** is lower in energy than **IM2** by 0.3 kcal mol^−1^. The distance between the iminol nitrogen and the carboxyl carbon is 3.265 Å in **IM3** (this distance was 3.191 Å in **IM2**). In the present study, we investigated the cyclization from **IM3**. The changes in the *φ*, *ψ*, and *χ*_1_ dihedral angles on going from **IM1** to **IM3** are again modest (Table 1). However, cumulative changes result in a total change by 40° in *ψ* when compared between **AM** and **IM3**. This is cancelled, however, by the change in the next cyclization step.

### 2.3. Cyclization to Form the Tetrahedral Intermediate

The cyclization from **IM3** to form the tetrahedral intermediate **TH** (Figure 10) proceeds via **TS5** (Figure 9) with a local activation barrier of 12.4 kcal mol^−1^. The relative energies of **TS5** and **TH** with respect to **AM** are 26.0 and 8.9 kcal mol^−1^, respectively. The distance of the forming N−C bond in **TS5** is 1.597 Å. Concomitantly with the N−C bond formation, a triple proton transfer occurs from the iminol oxygen to the carboxyl C=O oxygen mediated by the two water molecules, so that the resulting species (**TH**) is a *gem*-diol (a π-bond reorganization form O−C=N to O=C−N also accompanies). As may be seen from Figure 9, proton transfers from the water molecules are preceded by the other major changes in geometry. In **TH**, one water molecule forms hydrogen bonds to both of the OH groups in the *gem*-diol moiety. This water molecule acts as a catalyst in the next last step (dehydration to give the succinimide product **SI**). The other two water molecules form a hydrogen-bond bridge between the α-carbonyl group and one of the OH groups. Although no other pathways connecting **IM3** and **TH** were found, the possibility of multiple pathways may remain.

The *ψ* dihedral angle changes by 49° in the cyclization step (Table 1). However, the net change by 9° from **AM** is much less significant. On the other hand, a large change in *χ*_1_ by 36° is observed in the cyclization step. As a result, *χ*_1_ changes by 47° on going from **AM** to **TH**.

### 2.4. Dehydration of the Tetrahedral Intermediate

The dehydration of the *gem*-diol group of **TH** leading to the succinimide product **SI** (Figure 12) occurs via **TS6** (Figure 11) with a local activation barrier of 17.8 kcal mol^−1^. This step involves a double proton transfer mediated by the water molecule hydrogen-bonded to the *gem*-diol group, accompanying the scission of one of the two C−O bonds. The distance of the breaking C−O bond is 1.637 Å in **TS6**, which becomes 2.948 Å in **SI**.

The relative energy of **TS6** with respect to the reactant complex **AM** is 26.7 kcal mol^−1^, and this corresponds to the overall activation barrier of the entire reaction shown in Figure 1. Namely, the rate-determining step is predicted to be the last dehydration step. However, since the energy difference between **TS5** (the cyclization TS) and **TS6** is small (0.7 kcal mol^−1^), factors not considered in the present study may make the cyclization step to be rate-determining.

Experimentally, an Arrhenius plot has recently been reported for the succinimide formation in a human αA-crystallin peptide TVLDSGISEVR [15], from which the activation energy can be calculated to be 25.6 kcal mol^−1^. The calculated activation barrier agrees satisfactorily with this experimental value, suggesting that the water-catalyzed mechanism presented here, or one similar to it, actually operates under physiological conditions.

The rate constant is determined not only by the activation energy but also by the pre-exponential factor. The above Arrhenius plot gives a relatively low value of 3.1 × 10^10^ s^−1^ for the pre-exponential factor. This may partially be due to the requirement that three water molecules must align for the reaction to be initiated. On the other hand, we can expect some enthalpy−entropy compensation in the complex formation between the reactant and three water molecules [16].

In **TS6**, one water molecule acts as a proton-relay catalyst, while the other two water molecules are ‘spectators’. However, these spectators form relatively short hydrogen bonds of 1.687 and 1.697 Å in **TS6** (see Figure 11). Therefore, they may have some contribution to stabilizing **TS6**.

Experimentally, succinimide formation from Asp residues is endothermic [17]. However, **SI** was calculated to be 1.9 kcal mol^−1^ lower than the reactant complex **AM**. This may be due partly to an unbalanced description of hydration, because there are four explicit water molecules in **SI** while there are three in **AM**.

Changes in the *φ*, *ψ*, and *χ*_1_ dihedral angles are also modest in the dehydration step (Table 1). When compared between the reactant complex **AM** and the product complex **SI**, *φ* and *ψ* are almost unchanged, while *χ*_1_ changes by 62°. The latter large change in conformation can be a bottleneck when the reaction occurs in a structurally constrained environment.

## 3. Experimental Section

Figure 13 shows the model compound (Ace−Asp−Nme, where Ace = acetyl and Nme = NHCH_3_) used in the present study. This compound has previously been used in related computational studies by us [11,12] and Catak *et al.* [13,18]. Note that the Asp side chain is protonated. This is because only the protonated form is thought to undergo the nucleophilic attack by the backbone nitrogen to form the five-membered ring [2,11,12,17,19]. If we take a p*K*_a_ value of 3.9 for the Asp side-chain carboxyl group, only one of every 3,000 Asp residues is protonated at the physiological pH of 7.4 [2]. The entire reaction was investigated starting from a reactant complex in which a wire of three water molecules forms a bridge between the backbone carbonyl oxygen and the side-chain OH hydrogen of the Asp residue by hydrogen bonding.

As in our previous studies [11,12,20], all calculations were performed by the density functional theory (DFT) with the B3LYP functional and the 6-31+G(d,p) basis set using Spartan’08 [21]. Energy-minimum and transition state (TS) geometries were located in vacuum without any constraints. Vibrational frequency calculations were performed for all the optimized geometries to confirm them as energy minima (with no imaginary frequency) or TSs (with a single imaginary frequency) and to correct the relative energies for the zero-point energy (ZPE). The connectivity of all the optimized geometries as shown in Figure 1 was confirmed by intrinsic reaction coordinate (IRC) calculations and geometry optimizations. Successful IRC calculations were followed by full geometry optimization. When an IRC was not calculated successfully, the atoms were slightly displaced from the TS geometry along the transition vector (*i.e.*, the vibration with an imaginary frequency), followed by full geometry optimization.

## 4. Conclusions

By DFT calculations, we have been successful for the first time in showing a *single* reaction model which can *qualitatively* describe the *entire* multi-step process of succinimide formation from Asp residues. This model corresponds to the iminolization−cyclization−dehydration mechanism which we have recently proposed, and includes three catalytic water molecules which mediate multiple proton transfers. More specifically, the iminolization, cyclization, and dehydration steps are catalyzed by three, two, and one water molecule(s), respectively. The iminolization occurs in two steps where five protons are transferred along intramolecular and intermolecular hydrogen bonds. The cyclization and dehydration steps involve proton transfers along intermolecular hydrogen bonds only. The last dehydration step was predicted to be rate-determining (although the energy difference between the cyclization and dehydration TSs is small), and the calculated activation barrier agreed well with an experimental activation energy [15]. The results of the present and previous calculations strongly suggest that water molecules have crucial catalytic effects in the succinimide formation from aspartic acid residues in peptides and proteins. Although the effect of bulk water was not considered in the present study, the energetic results were rather satisfactory. This may suggest that the bulk water does not largely affect the energetics of the water-catalyzed succinimide formation from Asp residues. This point will be addressed in our future work.
